# Long-term outcomes after different treatments for gastric cancer with synchronous liver metastasis: A PRISMA systematic review and network meta-analysis

**DOI:** 10.1097/MD.0000000000029533

**Published:** 2022-06-24

**Authors:** Minghui Li, Bin Yang

**Affiliations:** a Department of General Surgery, Xinhua Hospital Affiliated to Dalian University, Dalian, PR China; b Department of General Surgery, 967 Hospital of the Joint Service Support Force of PLA, Dalian, PR China.

**Keywords:** clinical outcome, gastric cancer, gastric carcinoma, liver metastasis, network meta-analysis, synchronous, treatment

## Abstract

**Background:**

The treatment of gastric cancer (GC) with synchronous liver metastasis is still controversial. This systematic review and network meta-analysis was designed to evaluate the long-term outcomes after different treatments of GC with synchronous liver metastasis.

**Methods:**

Several electronic databases were searched to identify eligible studies updated on May 1, 2021. Studies assessing the overall survival (OS) after different treatments (including chemotherapy, interventional therapy, surgical therapy alone and adjunctive therapy after surgery) of GC with synchronous liver metastasis were included. Odds ratios with 95% confidence interval (CI) were calculated for survival variables.

**Results:**

A total of 15 studies including 4312 patients were included in this network meta-analysis. Adjunctive therapy after surgery performed better than surgery therapy alone (hazard ratio [HR] = 1.23, 95% credible interval [CrI]: 0.69–2.17), chemotherapy (HR = 1.18, 95%CrI: 0.71–1.95), and interventional therapy in terms of 1-year OS (HR = 2.03, 95%CrI: 1.22–3.37). In terms of 3-OS, adjunctive therapy after surgery showed better efficacy than surgery therapy alone (HR = 1.48, 95%CrI: 0.40–5.47), chemotherapy (HR = 1.27, 95%CrI: 0.37–4.35), and interventional therapy (HR = 3.16, 95%CrI: 0.73–13.63). For 5-OS, adjunctive therapy after surgery was superior to surgery therapy alone (HR = 1.74, 95%CrI: 0.08–37.76), chemotherapy (HR = 1.44, 95%CrI: 0.66–3.14), and interventional therapy (HR = 1.46, 95%CrI: 0.06–34.36). There were no statistical inconsistency and small-study effect existed in our network meta-analysis for 1-year, 3-year, or 5-year OS. Cluster ranking analysis performed with surface under the cumulative ranking showed adjuvant therapies after surgery (99.9, 96.7, 90.2) ranking higher than surgery therapy alone, chemotherapy, and interventional therapy for 1-year, 3-year, 5-year OS.

**Conclusion:**

The OS of adjuvant therapy after surgery was better than that of surgery therapy alone, chemotherapy, and interventional therapy. Adjuvant therapy after surgery is the most recommended therapy for people with GC with synchronous liver metastasis.

## 1 Introduction

Gastric cancer (GC) is the second leading cause of cancer death in the worldwide.^[1,2]^ Till now, surgery is still the main method of treatment for GC. However, some patients with GC are diagnosed with local late or distant metastasis and have lost the chance of surgery.^[3,4]^ Liver is a frequent site of distant metastasis from GC.^[5,6]^ Even for the patients with GC after radical surgical treatment, there are about 37% patients occur liver metastasis. Chemotherapy has been considered as the standard treatment method for metastatic GC.^[7]^ It has been reported that median survival time are 11.0 to 13.8 months for patients with unresectable and metastatic GC with chemotherapy treatment.^[8,9]^

Recently, several case series of liver resection for GC with synchronous liver metastasis has been reported. Liu et al.^[10]^ reported that simultaneous resection of both primary GC and synchronous hepatic metastasis may effectively prolong survival in the patients with gastric primary tumor and synchronous liver metastasis. Chen et al.^[11]^ reported that perioperative chemotherapy combined with surgery could improve prognosis of GC with synchronous liver metastasis. However, the efficacy of different treatments for GC with synchronous liver metastasis remains controversy.^[12,13]^ In our systematic review, we aim to evaluate all relevant evidence and perform a network meta-analysis to identify treatments of the patients with GC with synchronous liver metastasis. In order to include a broader spectrum of treatment approaches, randomized and non-randomized studies are both included in our meta-analysis.

## 2 Methods

### 2.1 Study selection strategy

The 2 authors independently retrieve the relevant database of EMbase, PubMed, Web of Science, the Cochrane Library, China database (CNKI, WanFang, and VIP). The retrieval time of the database is limited to May 1, 2021. The following key words were used: (“gastric cancer” or “stomach neoplasms” or “GC”) and (“hepatic metastasis” or “liver metastasis”) and (prognosis and “overall survival”) and synchronous.

### 2.2 Included criteria and excluded criteria

Studies should meet the following criteria: patients with GC with synchronous liver metastasis and without extrahepatic metastasis, included >20 patients, the outcomes of interest were survival and prognostic indicators, survival data for at least 1-year following surgical resection, and studies in English or Chinese.

Articles with the following exclusion criteria: the patients with extrahepatic metastasis, such as lung or peritoneal, without clinical data or survival data, case reports or review, data for repeat liver resection or metachronous liver metastasis, and duplicated publications.

### 2.3 Data extraction

Two individual authors extracted data from included studies. When there are different opinions, then agreement was reached by discussion. Information was extracted as follows: first author's name, published magazine, patient country, year of publication, age, gender, methods of treatment, number of patients, duration of treatment, survival outcome of 1-year, 3-year, or 5-year.

### 2.4 Assessment of methodological quality

Two authors separately assessed the quality of the retrieved studies and disagreements were resolved by discussion. Study quality was assessed using the Newcastle Ottawa Scale.

### 2.5 Ethical approval

This systematic review does not require ethical assessment because only indirect literature will be included and evaluated.

### 2.6 Statistical analysis

We used STATA 15 software (Stata Corp. College Station, TX) to perform network meta-analysis to combine evidence for 4 different methods of treatment for GC with synchronous liver metastasis. Odds ratios (ORs) with 95% confidence intervals (CIs) were calculated for survival. Surface under the cumulative ranking (SUCRA) probabilities was used to rank the effect of different treatments, and larger SUCRA scores reveal more effective treatments. The consistency between different comparisons was assessed by node-splitting method. Publication bias was tested by funnel plot. *P* value <.05 was considered statistically significant.

## 3 Results

### 3.1 Literature search and selection

**Figure 1:**
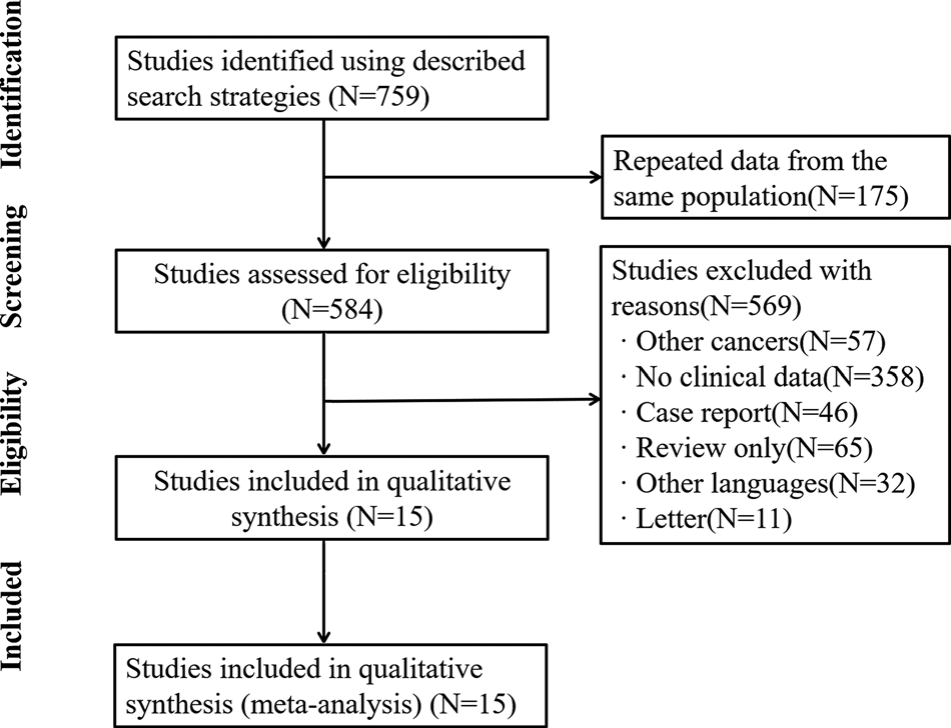
The Preferred reporting items for systematic reviews and meta-analysis (PRISMA) flow chart of the selection process to identify studies eligible for pooling.

After researching in the electronic database, 759 studies were retrieved. After selecting title and abstract, 175 duplicates were removed and 569 studies were excluded, including 65 reviews, 96 case reports, 11 letters, and 397 lack of clinical data (Fig. 1). Finally, 15 studies published from 2003 to 2018 were adopted in this network meta-analysis to conduct a prognosis comparison among the 4 different therapies.

### 3.2 Characteristics of the included studies

The characteristics of the included studies were presented in Table 1. A total of 15 studies including 4312 patients were included, 10 studies were come from China, 3 studies were come from Japan, 1 study was come from USA, and 1 was come from Italy. There were 3408 patients received chemotherapy, 157 patients received interventional therapy, 248 patients received surgical therapy alone, and 517 patients received adjunctive therapy after surgery (e.g., perioperative chemotherapy and adjuvant chemoradiation therapy). For the OS, 14 studies reported 1-year OS, 13 studies reported 3-year OS, and 9 studies included 5-year OS. The majority of the eligible studies were 2-arm or 3-arm trials while one of them was 4-arm trials.

**Table 1:** Main characteristics of the eligible studies.

No.	First author	Year	Cases no.	M/F	During	Country	RCT/Not	Treatment modality	NOS
1	Chen^[13]^	2013	114	71/43	2007.7–2012.10	China	Not	S and A	9
2	Sakamoto^[14]^	2007	37	29/8	1990–2005	Japan	Not	S and C	7
3	Li^[15]^	2015	49	–	2008.6–2011.12	China	Not	C and A	8
4	Qiu^[16]^	2013	25	22/3	1998.10–2009.12	China	Not	S and A	9
5	Koga^[17]^	2007	42	30/12	1985.1–2005.5	Japan	Not	S and A	6
6	Tomoki^[18]^	2017	34	24/10	1997.12–2015.12	Japan	Not	S and I	6
7	Wang^[19]^	2014	39	26/13	1996.1–2008.12	China	Not	S and C	7
8	Ministrini^[20]^	2018	144	94/50	1990–2017	Italy	Not	S and A	7
9	Picado^[12]^	2018	3175	-	2004–2014	USA	Not	C and A	9
10	Du^[21]^	2016	106	76/30	2008.6–2014.10	China	Not	S and C and A and I	7
11	Chen^[22]^	2007	31	22/9	1997.10–2006.10	China	Not	C and A	6
12	Hu^[23]^	2009	61	41/20	1999–2009	China	Not	C and I and A	6
13	Wang^[24]^	2003	91	-	1989.1–2001.7	China	Not	C and A	7
14	Jing^[25]^	2013	316	269/47	2001.5–2013.5	China	Not	C and A	7
15	Huang^[26]^	2009	48	30/18	1995.1–2004.12	China	Not	A and I	7

– = not report, A = adjunctive therapy after surgery, C = chemotherapy, F = female, I = interventional therapy, M = male, No. = number, S = surgical therapy.

**Figure 2:**
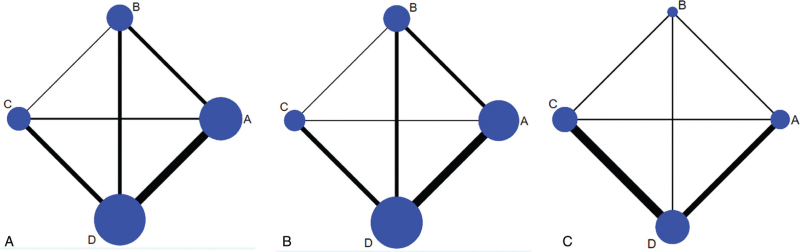
Network structure. (A) 1-year OS; (B) 3-year OS; (C) 5-year OS. The network plots show direct comparison of different treatments, with node size corresponding to the sample size. The thickness of solid lines corresponding to the number of included studies for direct comparison. A: chemotherapy; B: interventional therapy; C: surgical therapy alone; and D: adjunctive therapy after surgery. OS = overall survival.

The evidence network is showed in Fig. 2. Four direct comparisons about 1-year, 3-year, and 5-year OS were extracted from all included articles. The lines between 2 connected interventions show direct comparison of them, and the width of lines represents the number of included studies. The size of nodes represents the sample size of each intervention.

### 3.3 Network meta-analysis of OS

**Figure 3:**
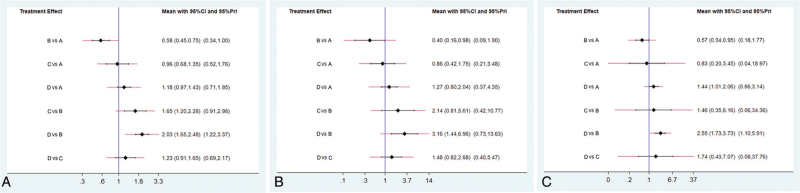
Forest plots for 1-year (A), 3-year (B), and 5-year OS (C). Hazard ratios (HRs) with 95% credible interval (Crls) indicate the relative efficacy. A: chemotherapy; B: interventional therapy; C: surgical therapy alone; and D: adjunctive therapy after surgery.

This network meta-analysis was conducted to reveal the OS of patients with GC with synchronous liver metastasis. As shown in Fig. 3A, adjunctive therapy after surgery performed better than surgery therapy alone (hazard ratio [HR] = 1.23, 95% credible interval [CrI]: 0.69–2.17), chemotherapy (HR = 1.18, 95%CrI: 0.71–1.95), and interventional therapy in terms of 1-year OS (HR = 2.03, 95%CrI: 1.22–3.37). Surgery therapy alone yielded better outcome than chemotherapy (HR = 0.96, 95%CrI: 0.52–1.76) and interventional therapy (HR = 1.65, 95%CrI: 0.91–2.98). In terms of 3-OS, adjunctive therapy after surgery revealed better efficacy compared with surgery therapy alone (HR = 1.48, 95%CrI: 0.40–5.47), chemotherapy (HR = 1.27, 95%CrI: 0.37–4.35), and interventional therapy (HR = 3.16, 95%CrI: 0.73–13.63) (Fig. 3B). The similar results were revealed when comparing 5-OS in Fig. 3C, with adjunctive therapy after surgery superior to surgery therapy alone (HR = 1.74, 95%CrI: 0.08–37.76), chemotherapy (HR = 1.44, 95%CrI: 0.66–3.14), and interventional therapy (HR = 1.46, 95%CrI: 0.06–34.36).

### 3.4 Inconsistency test and publication bias

**Figure 4:**
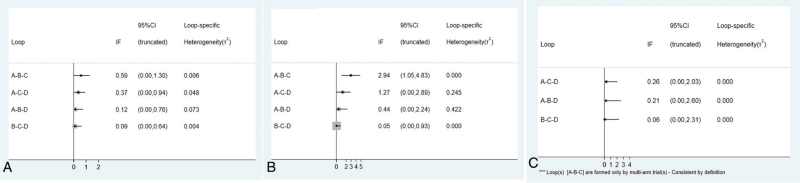
Inconsistency test for direct and indirect comparisons. (A) 1-year OS; (B) 3-year OS; (C) 5-year OS. A: chemotherapy; B: interventional therapy; C: surgical therapy alone; and D: adjunctive therapy after surgery. OS = overall survival.

**Figure 5:**
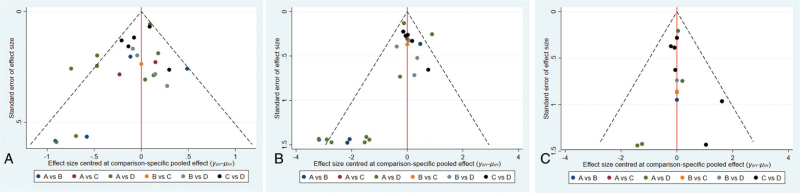
Funnel plots assessment of publication bias of all included studies. (A) 1-year OS; (B) 3-year OS; (C) 5-year OS. A: chemotherapy; B: interventional therapy; C: surgical therapy alone; and D: adjunctive therapy after surgery.

This network meta-analysis of 1-year OS was composed of 4 triangular loops (the A-B-C, A-C-D, A-B-D, B-C-D loop). The inconsistency factor (IF) of each loop was 0.59 (95% CI: 0.00–1.30), 0.37 (95% CI: 0.00–0.94), 0.12 (95% CI: 0.00–0.76), 0.09 (95% CI: 0.00–0.64). The 95% CI of IF reached 0, indicating that no statistical inconsistency existed (Fig. 4A). The funnel plot was roughly symmetrical, demonstrating that no small-study effect existed in our network meta-analysis (Fig. 5A).

The results of IF of each loop 3-OS shown in Fig. 4B. The IF of each loop was 2.94 (95% CI: 1.05–4.83), 1.27 (95% CI: 0.00–2.89), 0.44 (95% CI: 0.00–2.24), 0.05 (95% CI: 0.00–0.93). The 95% CI of IF reached 0, indicating that no statistical inconsistency existed. The funnel plot was roughly symmetrical, demonstrating that no small-study effect existed in our network meta-analysis (Fig. 5B).

This network meta-analysis of 5-year OS was composed of 3 triangular loops (the A-C-D, A-B-D, B-C-D loop). The results of IF of each loop 5-OS shown in Fig. 4C. The IF of each loop was 0.26 (95% CI: 0.00–2.03), 0.21 (95% CI: 0.00–2.60), 0.06 (95% CI: 0.00–2.31). The 95% CI of IF reached 0, indicating that no statistical inconsistency existed. The funnel plot was roughly symmetrical, demonstrating that no small-study effect existed in our network meta-analysis (Fig. 5C).

### 3.5 Cluster ranking

**Figure 6:**
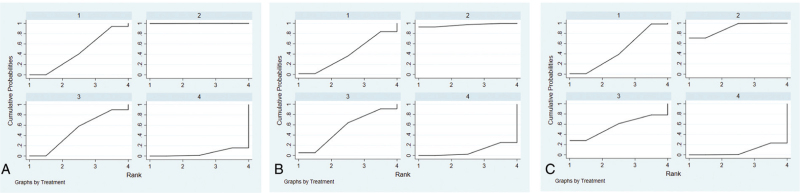
Surface under the cumulative ranking curve for chemotherapy, interventional therapy, surgery therapy alone, and adjuvant therapies after surgery. (a) 1-year OS; (b) 3-year OS; (c) 5-year OS. OS = overall survival.

Ranking analysis performed with SUCRA and showed adjuvant therapies after surgery ranking higher than surgery therapy alone, chemotherapy, and interventional therapy. Adjuvant therapies after surgery (99.9, 96.7, 90.2) ranked first for 1-year (Fig. 6A), 3-year (Fig. 6B), 5-year OS (Fig. 6C). The other 3 treatments were ranked as follows: surgery therapy alone (49.4, 53.4, 55.8), chemotherapy (44.9, 40.5, 46.2), and interventional therapy (5.7, 9.3, 7.9).

## 4 Discussion

Liver metastasis is one of the common organs of distant metastasis of GC, the 5-year survival rate of liver metastasis is as low as 6% to 13.1%.^[27,28]^ Till now, concentrating on treatment for liver metastasis of GC, there is still no consistent standardized therapeutic regimen around the world. Chemotherapy is still the main treatment for GC with synchronous liver metastasis.^[9]^ In recent years, liver resection for GC with synchronous liver metastasis has been investigated. The optimal treatment for GC with synchronous liver metastasis remains controversial. The adjunctive therapy after surgery, surgery therapy alone, chemotherapy, and interventional therapy are the 4 main treatment methods used.^[12,13–26]^ Recently, some studies have compared these methods using traditional meta-analysis.^[27,28]^ To the best of our knowledge, all studies focused on the comparison between 2 treatments.^[29,30]^ We carried out a network meta-analysis, comparing the long-term outcomes after different managements for GC with synchronous liver metastasis even if there was no direct comparison.

The current network meta-analysis was based on 15 studies including 4312 patients and compared the long-term outcomes after different managements for GC with synchronous liver metastasis. In our results, we found the OS of adjuvant therapies after surgery was better than surgery therapy alone, chemotherapy, and interventional therapy. Furthermore, ranking probability indicated that adjuvant therapies after surgery was the most likely to result in a better treatment of GC with synchronous liver metastasis.

In recent decades, multimodality including chemotherapy, interventional therapy, surgical therapy or some of them has been used to improve treatment outcomes of GC with synchronous liver metastasis. As non-surgical treatments, such as systemic or hepatic artery infusion chemotherapy, could not achieve satisfactory results.^[31]^ Considering the possibility to receive radical surgery of metastasis, leaving enough residual liver and the tolerance of patient, only few GC patients with liver metastasis had a chance of radical surgery,^[19,32]^ so that the efficiency of surgery upon prognosis has not been still reached a completely consistent. Liver metastasis surgical resection cannot be suitable for all GC patients with synchronous liver metastasis, but the prognosis prolonging results have been demonstrated. Recent years, some authors reported that the patients could harvest a significant better survival when they receiving the resection of liver metastasis.^[19,33]^ So for GC patients with liver metastasis, we suggested that radical surgery for both primary and metastatic tumor should be performed if indications can be appropriated to perform, and the radical surgery treatment may bring survival benefit for GC patients with liver metastasis.

The characteristics associated with the survival of GC with liver metastasis have not been comprehensively identified. Previous studies have identified several prognostic factors after liver resection for GC with synchronous liver metastasis, including number of liver metastasis, unilobar lesions, negative margin resection, and adjunctive therapy after surgery.^[12,34]^ The adjunctive therapy after surgery is often considered as an important favorable prognostic factor.^[30]^ In our results, we found that the OS of adjuvant therapies after surgery was better than surgery therapy alone, chemotherapy, and interventional therapy. Furthermore, ranking probability indicated that adjuvant therapies after surgery was the most likely to result in a better treatment of GC with synchronous liver metastasis. We recommend more personally tailored multimodality treatment approaches (surgery + adjuvant therapies) in GC patients with synchronous liver metastasis. Further multi-center study with a larger population is required to confirm the results, and we hope that our results of this meta-analysis can provide a reference for clinicians.

There are several limitations in our network meta-analysis. First, some of our included studies are high-selective, and the complications were not reported in most of the studies. Second, the surgical excision scope, surgical techniques, and surgical effect are different in the eligible studies. Third, we only included the articles written in English and Chinese, and the included articles were retrospective analysis, therefore, the bias is not neglected. Furthermore, some mixed factors, such as gender, age, different adjuvant therapies after surgery, were not included in our study.

In conclusion, our network meta-analysis showed that long-term survival rate of adjuvant therapy after surgery was better than that of surgery therapy alone, chemotherapy, and interventional therapy. Adjuvant therapy after surgery is the most recommended treatment for GC people with synchronous liver metastasis. However, adverse effects of this therapy are concerned because of the absence of clinical data, its safety is still unclear. More studies, especially randomized control trials, are needed to perform to confirm this conclusion.

## Author contributions

**Conceptualization:** Minghui Lia, Bin Yang.

**Data curation:** Minghui Lia, Bin Yang.

**Formal analysis:** Minghui Lia, Bin Yang.

**Investigation:** Minghui Lia, Bin Yang.

**Methodology:** Minghui Lia, Bin Yang.

**Software:** Minghui Lia, Bin Yang.

**Supervision:** Minghui Lia, Bin Yang.

**Writing – original draft:** Minghui Lia.

**Writing – review & editing:** Bin Yang.

## References

[1] Topi S Santacroce L Bottalico L. Gastric cancer in history: a perspective interdisciplinary study. Cancers (Basel) 2020;12:264. 10.3390/cancers12020264 PMC7072612 31978985

[2] Salati M Orsi G Smyth E. Gastric cancer: translating novels concepts into clinical practice. Cancer Treat Rev 2019;79:101889. 31445415 10.1016/j.ctrv.2019.101889

[3] Macrì A Morabito F. The use of intraperitoneal chemotherapy for gastric malignancies. Expert Rev Anticancer Ther 2019;19:879–88. 31544548 10.1080/14737140.2019.1671189

[4] Lee JH Kim HI Kim MG. Recurrence of gastric cancer in patients who are disease-free for more than 5 years after primary resection. Surgery 2016;159:1090–8. 26747230 10.1016/j.surg.2015.11.002

[5] Sun Z Zheng H Yu J. Liver metastases in newly diagnosed gastric cancer: a population-based study from SEER. J Cancer 2019;10:2991–3005. 31281476 10.7150/jca.30821 PMC6590027

[6] Song JC Ding XL Zhang Y. Prospective and prognostic factors for hepatic metastasis of gastric carcinoma: a retrospective analysis. J Cancer Res Ther 2019;15:298–304. 30964101 10.4103/jcrt.JCRT_576_17

[7] Nakayama N Ishido K Chin K. A phase I study of S-1 in combination with nab-paclitaxel in patients with unresectable or recurrent gastric cancer. Gastric Cancer 2017;20:350–7. 27189323 10.1007/s10120-016-0614-4

[8] Cavanna L Bodini FC Stroppa EM. Advanced gastric cancer with liver and lymph node metastases successfully resected after induction chemotherapy with docetaxel, cisplatin and 5-fluorouracil. Chemotherapy 2014;60:224–7. 25872022 10.1159/000375156

[9] Ina K Furuta R Kataoka T. Chemo-immunotherapy using Lentinan for the treatment of gastric cancer with liver metastases. Med Sci (Basel) 2016;4:08. 10.3390/medsci4020008 PMC5635777 29083372

[10] Liu Q Bi JJ Tian YT. Outcome after simultaneous resection of gastric primary tumour and synchronous liver metastases: survival analysis of a single-center experience in China. Asian Pac J Cancer Prev 2015;16:1665–9. 25743789 10.7314/apjcp.2015.16.4.1665

[11] Chen L Song MQ Lin HZ. Chemotherapy and resection for gastric cancer with synchronous liver metastases. World J Gastroenterol 2013;19:2097–103. 23599631 10.3748/wjg.v19.i13.2097 PMC3623989

[12] Picado O Dygert L Macedo FI. The role of surgical resection for stage IV gastric cancer with synchronous hepatic metastasis. J Surg Res 2018;232:422–9. 30463751 10.1016/j.jss.2018.06.067

[13] Kataoka K Kinoshita T Moehler M. Current management of liver metastases from gastric cancer: what is common practice? New challenge of EORTC and JCOG. Gastric Cancer 2017;20:904–12. 28150070 10.1007/s10120-017-0696-7

[14] Sakamoto Y Sano T Shimada K. Favorable indications for hepatectomy in patients with liver metastasis from gastric cancer. J Surg Oncol 2007;95:534–9. 17219383 10.1002/jso.20739

[15] Li Z Fan B Shan F. Gastrectomy in comprehensive treatment of advanced gastric cancer with synchronous liver metastasis: a prospectively comparative study. World J Surg Oncol 2015;13:212. 26126412 10.1186/s12957-015-0627-1 PMC4491213

[16] Qiu JL Deng MG Li W. Hepatic resection for synchronous hepatic metastasis from gastric cancer. Eur J Surg Oncol 2013;39:694–700. 23579173 10.1016/j.ejso.2013.03.006

[17] Koga R Yamamoto J Ohyama S. Liver resection for metastatic gastric cancer: experience with 42 patients including eight long-term survivors. Jpn J Clin Oncol 2007;37:836–42. 17928333 10.1093/jjco/hym113

[18] Ryu T Takami Y Wada Y. Oncological outcomes after hepatic resection and/or surgical microwave ablation for liver metastasis from gastric cancer. Asian J Surg 2019;42:100–5. 29254868 10.1016/j.asjsur.2017.09.005

[19] Wang W Liang H Zhang H. Prognostic significance of radical surgical treatment for gastric cancer patients with synchronous liver metastases. Med Oncol 2014;31:258. 25260807 10.1007/s12032-014-0258-3

[20] Ministrini S Solaini L Cipollari C. Surgical treatment of hepatic metastases from gastric cancer. Updates Surg 2018;70:273–8. 29845462 10.1007/s13304-018-0536-2

[21] Du Jin-ke Li Dong-ming. Comparison of clinical efficacy of different treatment methods for synchronous liver metastasis from gastric cancer and regression analysis of prognostic risk factors. Chin J Curr Adv Gen Surg 2016;19:691–4. 24577763

[22] Chen sheng-lin Wang Bing Yu shui-ping. Surgical treatment of 31 cases of hepatic metastasis tumor from gastric carcinoma. Fu Bu Wai Ke 2007;20:97–8.

[23] Hu W-D Xu M-R Cheng J-P. Treatment and prognosis for gastric cancer with hepatic metastases after operation. Jiangsu Med J 2009;35:428–9.

[24] Meng-chun W Hong-qian X Dong-kui X. Surgical treatment of hepatic metastasis of gastric carcinoma. Chin J Gen Surg 2003;18:217–8.

[25] Jianfeng J Kaichun W. Clinical study on the factors influencing the prognosis of hepatic metastasis in gastric carcinoma and different treatment methods. China Modern Doctor 2013;51:47–50.

[26] Huang Z-H Yan G Ge X-G. Surgical effect and prognosis analysis in 48 cases of combined over metastasis of gastric cancer. Suzhou Univ J Med Sci 2009;29:979–80.

[27] Cui JK Liu M Shang XK. Hepatectomy for liver metastasis of gastric cancer: a meta-analysis. Surg Innov 2019;26:692–7. 31267829 10.1177/1553350619856491

[28] Shen W Li J Cui J. Meta-analysis of prognosis after surgical treatment in gastric cancer patients with liver metastasis. Zhonghua Wei Chang Wai Ke Za Zhi 2014;17:128–32. 24577766

[29] Tiberio GA Ministrini S Gardini A. Factors influencing survival after hepatectomy for metastases from gastric cancer. Eur J Surg Oncol 2016;42:1229–35. 27134189 10.1016/j.ejso.2016.03.030

[30] Gao J Wang Y Li F. Prognostic nutritional index and neutrophil-to- lymphocyte ratio are respectively associated with prognosis of gastric cancer with liver metatasis undergoing and without hepatectomy. Biomed Res Int 2019;2019:4213623. 31687389 10.1155/2019/4213623 PMC6800959

[31] Kunieda K Saji S Sugiyama Y. Evaluation of treatment for synchronous hepatic metastases from gastric cancer with special reference to long-term survivors. Surg Today 2002;32:587–93. 12111514 10.1007/s005950200106

[32] Xiao Y Zhang B Wu Y. Prognostic analysis and liver metastases relevant factors after gastric and hepatic surgical treatment in gastric cancer patients with metachronous liver metastases: a population-based study. Ir J Med Sci 2019;188:415–24. 30062399 10.1007/s11845-018-1864-4

[33] Takemura N Saiura A Koga R. Long-term outcomes after surgical resection for gastric cancer liver metastasis: an analysis of 64 macroscopically complete resections. Langenbecks Arch Surg 2012;397:951–7. 22615045 10.1007/s00423-012-0959-z

[34] Tatsubayashi T Tanizawa Y Miki Y. Treatment outcomes of hepatectomy for liver metastases of gastric cancer diagnosed using contrast-enhanced magnetic resonance imaging. Gastric Cancer 2017;20:387–93. 27155874 10.1007/s10120-016-0611-7

